# SETD7 Regulates the Differentiation of Human Embryonic Stem Cells

**DOI:** 10.1371/journal.pone.0149502

**Published:** 2016-02-18

**Authors:** Julio Castaño, Cristina Morera, Borja Sesé, Stephanie Boue, Carles Bonet-Costa, Merce Martí, Alicia Roque, Albert Jordan, Maria J. Barrero

**Affiliations:** 1 Center for Regenerative Medicine in Barcelona, (CMRB), Barcelona, 08003, Spain; 2 Biomedical Research Networking Center in Bioengineering, Biomaterials and Nanomedicine (CIBER-BBN), Madrid, 28029, Spain; 3 Institut de Biologia Molecular de Barcelona (IBMB-CSIC), Barcelona, 08028, Spain; 4 Departamento de Bioquímica y Biología Molecular, Facultad de Biociencias, Universidad Autónoma de Barcelona, Cerdanyola, 08193, Barcelona, Spain; 5 Spanish National Cancer Research Center (CNIO), Madrid, 28029, Spain; Wellcome Trust Centre for Stem Cell Research, UNITED KINGDOM

## Abstract

The successful use of specialized cells in regenerative medicine requires an optimization in the differentiation protocols that are currently used. Understanding the molecular events that take place during the differentiation of human pluripotent cells is essential for the improvement of these protocols and the generation of high quality differentiated cells. In an effort to understand the molecular mechanisms that govern differentiation we identify the methyltransferase SETD7 as highly induced during the differentiation of human embryonic stem cells and differentially expressed between induced pluripotent cells and somatic cells. Knock-down of SETD7 causes differentiation defects in human embryonic stem cell including delay in both the silencing of pluripotency-related genes and the induction of differentiation genes. We show that SETD7 methylates linker histone H1 in vitro causing conformational changes in H1. These effects correlate with a decrease in the recruitment of H1 to the pluripotency genes *OCT4* and *NANOG* during differentiation in the SETD7 knock down that might affect the proper silencing of these genes during differentiation.

## Introduction

The generation of specialized cell types from human pluripotent cells in the laboratory can provide an unlimited source of cells and tissues useful for transplantation and therefore holds a great promise for regenerative medicine (Reviewed in [1]). Successful therapies depend on the generation of functional cell types that have enough plasticity to survive and repopulate the damaged tissues with a low risk of forming tumors [2]. In order to achieve these goals the current protocols used to differentiate cells will need to be improved and the quality of the differentiated products more strictly evaluated. A recent study comparing the similarity of *in vitro* and *in vivo* differentiated cells highlights the existence of significant differences both at the level of gene expression and chromatin marks [3], confirming that improved in vitro differentiation methods are needed to obtain cells similar to their *in vivo* counterparts. Therefore, understanding the molecular mechanisms that take place during differentiation appears essential for the development of optimal differentiation protocols.

Important advancements in the stem cell field include the generation of human induced pluripotent stem cells (iPSCs) from somatic cells [4] which show similar properties to human embryonic stem cells (ESCs). While iPSCs cells can provide an invaluable source of cells for autologous transplantation, their safety for use in the clinic is still unclear [5]. In order to assess the potential risks of using reprogrammed cells for therapy a closer look at the mechanisms of reprogramming and their consequences is warranted.

In an effort to identify critical factors involved in determining cell identity we previously compared the expression patterns of pluripotent and somatic cells [6]. We described a network of factors that are predominantly expressed in pluripotent human cells, encompassing factors that had been previously used to reprogram cells such as OCT4, SOX2, NANOG, LIN28 or SALL4, components of signal transduction pathways such as TGDF1, FGFR2, FGFR3 and NODAL, and the chromatin-related proteins PRDM14, TET1, JARID2, DNMTs and CBX2. Importantly, we also identified a network of genes that were preferentially expressed in differentiated cells, including the histone variant H2AFY that plays critical roles in preserving the identity of somatic cells [7,8].

Among the factors that we found upregulated in somatic cells compared to pluripotent cells we noticed the protein lysine methyltransferase SETD7 (also called SET7/9 or KMT7). SETD7 was initially described as a histone methyltransferase able to mediate the monomethylation of histone H3 at lysine 4 (H3K4me1) in vitro [9]. However, the fact that it cannot efficiently methylate nucleosomal substrates [9,10] suggests that its physiological substrate in vivo might be different than histone H3. Accordingly, numerous non-histone targets have been described for SETD7, including p53 [11], ERα [12], p65 [13], STAT3 [14], pRB [15], SIRT1[16], DNMT1 [17], FOXO3 [18], SUV39H1 [19], E2F1 [20], AR [21], FXR [22], PCAF [23], PARP1 [24] and TAF10 [25] that are potential mediators of SETD7 effects.

Here, we have confirmed that SETD7 is expressed at very low levels in human pluripotent cells and strongly induced during differentiation. We have identified novel SETD7 interaction partners in differentiated cells. Among these partners we describe that linker histone H1 is methylated by SETD7. This methylation is likely to lead to structural changes that modulate the affinity of histone H1 for chromatin during human pluripotent cells differentiation contributing to orchestrate the changes in gene expression that take place during this process.

## Materials and Methods

### Cell culture

Human embryonic stem cell lines used in this study were previously published; ES[4] and ES[2] (described in [26]) and KiPSCs (described in [27]). For viral infection cells were grown in matrigel coated plates, in the presence of irradiated MEF’s conditioned HES media (Knock Out DMEM supplemented with 20% KO serum replacement, 1X MEM non essential amino acids, 2mM L-glutamine and 50μM β-mercaptoethanol) supplemented with 10ng/ml FGF and subcultured as aggregates using trypsin.

For differentiation studies using the SETD7 inhibitor pluripotent cells were cultured in matrigel coated plated using mTeSR1 media (STEMCELL Technologies) and subcultured as aggregates using dispase. Keratinocytes and fibroblasts were cultured as previously described [28].

### Lentiviral vectors and viral production

pLKO.1-puro lentiviral vectors containing different shRNAs against human SETD7 were purchased from SIGMA TRCN0000078628 (sh28), TRCN0000078629 (sh29), TRCN0000078630 (sh30), TRCN0000078631 (sh31), TRCN0000078632 (sh32). Viruses were produced as previously described [29]. For FLAG tagged SETD7 over expression we used the lentiviral vector pWPI (http://tronolab.epfl.ch) (Addgene plasmid 12254).

### In vitro differentiation of pluripotent cells

Lentiviral infected cells were trypsinized into a single cell suspension and resuspended in MEF’s conditioned HES media. Embryoid body (EB) formation was induced by seeding 100,000 cells in each well of 96-well v-bottom, low attachment plates and centrifuging the plates at 950g for 5 min to aggregate the cells. After 3 days the embryoid bodies were transferred to 0.1% gelatin-coated dishes and cultured in differentiation medium (Knock out DMEM supplemented with 20% fetal bovine serum, 1X MEM non essential amino acids, 2mM L-glutamine and 50μM β-mercaptoethanol) up to 20 days. The medium was changed every four days.

For the SETD7 inhibitor PFI-2 treatment cells were maintained in mTeSR1 media, detached using dispase and cultured in suspension as agregates in low attachment plates for 48 hours. Then the formed embryoid bodies were transferred to 0.1% gelatin-coated slide flasks and cultured in differentiation medium (Knock out DMEM supplemented with 20% fetal bovine serum, 1X MEM non essential amino acids, 2mM L-glutamine and 50μM β-mercaptoethanol) and presence of vehicle (DMSO) or PFI-2 up to 7 days. The medium was changed every four days.

### Real time PCR and microarray analysis

Total RNA was isolated using TRIZOL and 1 μg was used to synthesize cDNA using the Invitrogen Cloned AMV First-Strand cDNA synthesis kit. One μl of the reaction was used to quantify gene expression by qPCR. Oligonucleotides for amplification of endogenous pluripotency factors and differentiation genes were previously described [27]. Oligonucleotides for p21 and SETD7 amplification were the following (F, forward; R, reverse): SETD7.F 5’TTCACTCCAAACTGCATCTACGA3’; SETD7.R 5’GCATTTGATGGGCCCAAA3’; P21.F 5’TGGAGACTCTCAGGGTCGAAA3’; P21.R 5’GCGTTTGGAGTGGTAGAAATCTG3’.

For microarray analysis total RNA was isolated using the RNAeasy kit from Qiagen and analyzed using the Affymetrix Human U133 2.0 Plus array. The GeneChip microarray processing was performed by the Functional Genomics Core in the Institute for Research in Biomedicine (Barcelona, Spain) according to the manufacturer's protocols (Affymetrix) as described previously [30]. The amplification and labeling were processed as indicated in the Nugen protocol with 25 ng starting RNA. For each sample, 3.75 mg single-stranded DNA was labeled and hybridized to the chips. Expression signals were scanned on an Affymetrix GeneChip Scanner (7G upgrade). The data extraction was done by the Affymetrix GCOS software v.1.4. Raw data were normalized using the gcRMA algorithm implemented in R. Heatmaps were generated using GENE-E (http://www.broadinstitute.org/cancer/software/GENE-E) and box plots using BoxPlotR (http://boxplot.tyerslab.com/).

For GSEAPreranked [31] genes were pre-ranked according to fold change for each treatment obtained in the RNA-seq analysis, setting ‘gene set’ as the permutation method and with 1000 permutations.

Data has been deposited at GEO under accession number GSE24768.

### Western blot analysis

Whole cell extracts were isolated using RIPA buffer and 30 μg protein was analyzed by western blot using specific antibodies against OCT4 (Santa Cruz sc-5279), SOX2 (Neuromics GT15098), α-1-Fetoprotein (AFP; Dako, A0008), SETD7 (Millipore 07–314), TUBA (Santa Cruz sc-135592), ACTB (Sigma AV40173) or FLAG (Sigma M2).

### Cell Cycle Analyses

Cell cycle analyses were performed using Click-iT® EdU Alexa Fluor® 488 Flow Cytometry Assay Kit (Invitrogen) according to manufacturer instructions.

### Immunofluorescence

Cells were grown on plastic cover slide chambers and fixed with 4% paraformaldehyde at room temperature for 20 min. The immunodetection was performed with TBS-0.2%triton X-100 for permeabilization, primary antibodies were incubated at 4°C overnight and secondary antibodies at 37°C for 2h. Antibodies used were OCT4 (sc-5279 from Santa Cruz) and SOX2 (PA1-16968 from ABR). Secondary antibodies used were all cyanine conjugated from Jackson (all 1:400). Images were taken using Leica SP5 AOBS confocal microscope.

### Chromatin immunoprecipitation

The chromatin immunoprecipitation (ChIP) assays were performed according to the Millipore protocol. Briefly, 1 million human ESCs were used for each immunoprecipitation. Cells were fixed using 1% formaldehyde, harvested, resuspended in ChIP lysis buffer (1% SDS, 10mM EDTA, 50mM Tris-HCl, pH 8.1) and sonicated using the Branson Digital Sonifier to generate fragments of 150 to 500 bp. Soluble chromatin was diluted 8 fold in ChIP RIPA buffer (10 mM Tris–HCl, pH 7.5, 140 mM NaCl, 1 mM EDTA, 0.5 mM EGTA, 1% Triton X-100, 0.1% SDS, 0.1% Na-deoxycholate) and incubated with Dynabeads Protein A (Invitrogen) coupled to normal rabbit IgG or specific antibodies from Millipore against H3K4me2 (07–030), H3K27me3 (07–449) or H1 (clone AE-4). After incubation, the immunocomplexes were washed sequentially with Low Salt Wash Buffer (0.1% SDS, 1% Triton X-100, 2mM EDTA, 20mM Tris-HCl, pH 8.1, 150mM NaCl), High Salt Wash Buffer (0.1% SDS, 1% Triton X-100, 2mM EDTA, 20mM Tris-HCl, pH 8.1, 500mM NaCl), LiCl Wash Buffer (0.25M LiCl, 1% NP40, 1% deoxycholate, 1mM EDTA, 10mM Tris-HCl, pH 8.1) and TE. Immunocomplexes were eluted in ChIP elution buffer (1%SDS, 0.1M NaHCO3) and the crosslinking was reverted overnight at 65°C. Samples were treated with Proteinase K and RNase A and DNA was extracted using the Qiagen PCR purification kit. Purified chromatin was quantified using qPCR and the following oligonucleotides (F, forward; R, reverse): SETD7Pro.F 5’ ACCGGCGCTGGGTGAT3’; SETD7Pro.R 5’CCTGTAATACCCTCTCTTTGTTATCGA3’. Oligonucleotides for amplification of OCT4, NANOG and GAPDH regulatory regions were previously reported [8].

### Identification of SETD7 interacting proteins

Nuclear extract from 400x10^6^ HeLa cells transduced with empty vector or with FLAG:SETD7 overexpressing vector was prepared as previously described [32]. Extract were adjusted to 300 mM salt and 0.1% NP40 and incubated with ANTI-FLAG M2 affinity gel (Sigma). Beads were extensively washed with BC300 (20 mM Tris⋅Cl, pH 7.5, 20% glycerol, 0.2 mM EDTA and 300 mM KCl) containing 0.1% NP40. Proteins were eluted using 0.3 mg/ml of FLAG peptide, precipitated using trichloroacetic acid and resolved on SDS-PAGE gels or directly processed for mass spectrometry. Two independent immunoprecipitations were used for each method.

### In vitro methylation assays

H1 variants constructs were generated by PCR amplification from lentiviral expression vectors [33] and cloned into 6His-pET11d. Mutations were introduced by overlap extension. Expression plasmid for His-tagged SETD7 was previously described [34]. His-tagged proteins were expressed in *E*.*coli* and purified over Ni-NTA agarose. Calf thymus H1 was purchased from Santa Cruz Biotechnology. For expression of GST-H3 tail the H3 tail (residues 1–41) was cloned into the vector pGEX4T2 and purified using glutathione-Sepharose. Recombinant H3 was expressed in *E*.*coli* and purified as previously described [35]. Purified PRMT1 was a gift from S. Malik [36].

*In vitro* methylation assays were performed in a total volume of 20 μl containing 50 mM Tris-HCl (pH 9.0), 1 mM phenylmethylsulfonyl fluoride (PMSF), 0.5 mM dithiothreitol (DTT), 1 μl [3H]SAM (15 Ci/mmol; PerkinElmer), 2 μg of substrate and 200 ng of enzyme at 30°C for 2 hours. Products were resolved by SDS-PAGE, stained with Coomassie and analyzed by fluorography. Alternatively, 20μM SAM (SIGMA) was used and samples subjected to mass spectrometry.

### Mass spectrometry analysis of methylated H1

After in vitro methylation, sample was subjected to chromatography on a BioSuite pPhenyl 1000 column (Waters) (10 μm RPC, 4.6 × 75 mm), using a linear gradient of 5% to 80% B in 60 min (A = 0.1% FA in H2O, B = 0.1% FA in CH3CN) at a flow rate of 1 ml/min. The LC eluent was coupled to a LCT-Premier XE mass spectrometer (Waters-Microness) provided with an ESI source. Data was acquired with MassLynx software, V4.1. SCN639 (Waters Inc.). Charged species were deconvoluted to zero charged average mass with MaxEnt 1 algorithm (Waters Inc.)

### Infrared spectroscopy

FTIR spectra of unmethylated and methylated human histone H1.4 were measured at 5 mg/ml in 10 mM HEPES, pH 7.0 plus 140 mM NaCl in D2O medium. Measurements were performed on a FT600 Bio-Rad spectrometer equipped with a MCT detector, using a demountable liquid cell with calcium fluoride windows and 50 μm spacers. Typically, 1000 scans for each background and sample were collected and the spectra were obtained with a nominal resolution of 2 cm-1, at 22°C. DNA-protein complexes contained the appropriate amount of DNA for each protein/DNA ratio (w/w). The DNA contribution to the spectra of the complexes with the H1.4 species was subtracted as described [37]. Data treatment and band decomposition of the original amide I’ band was performed as previously described [38] using GRAMS 9.0 software.

### In vivo methylation of Histone extracts

HeLa cells were incubated in growth medium Dulbecco’s Modified Eagle’s Medium (DMEM), 10% Fetal Bovine Serum (FBS), 100 μg/ml cycloheximide, 40 μg/ml chloroamphenicol and 50 μl of L-[methyl-3H] methionine (1mCi/ml) for 3 hours at 37°C. After that the histone fraction was isolated by acid extraction and resolved by SDS-PAGE. Proteins were visualized by Coomassie blue staining, followed by autoradiography.

### Source of public data

Public data in the human embryonic cell line H1 was generated by the ENCODE project [39]. Data on H3K27me3 (GSM733748), H3K4me2 (GSM733670) and H3K4me3 (GSM733657) was generated at the Bernstein laboratory (Broad Institute). Data on Pol II occupancy (GSM822300) was generated at the Iyer laboratory (University of Texas at Austin). Data on DNA methylation (GSM683770) was generated at the Myers laboratory (Hudson Alpha Institute for Biotechnology). Data was displayed in the UCSC Genome Browser.

### Statistical methods

Benjamini P-values for gene ontology functional annotation were obtained using the DAVID analysis tool (http://david.abcc.ncifcrf.gov/). The significance of the differences in cell cycle distribution, H1 occupancy and gene expression at different days of differentiation were analyzed using the Student’s t test.

### Ethical statement

This study was done in accordance with Spanish laws and regulations regarding the manipulation of human pluripotent cells and approved by the Spanish competent authorities (Comisión de Seguimiento y Control de la Donación de Células y Tejidos Humanos del Instituto de Salud Carlos III).

## Results

### SETD7 is preferentially expressed in differentiated cells

**Fig 1:**
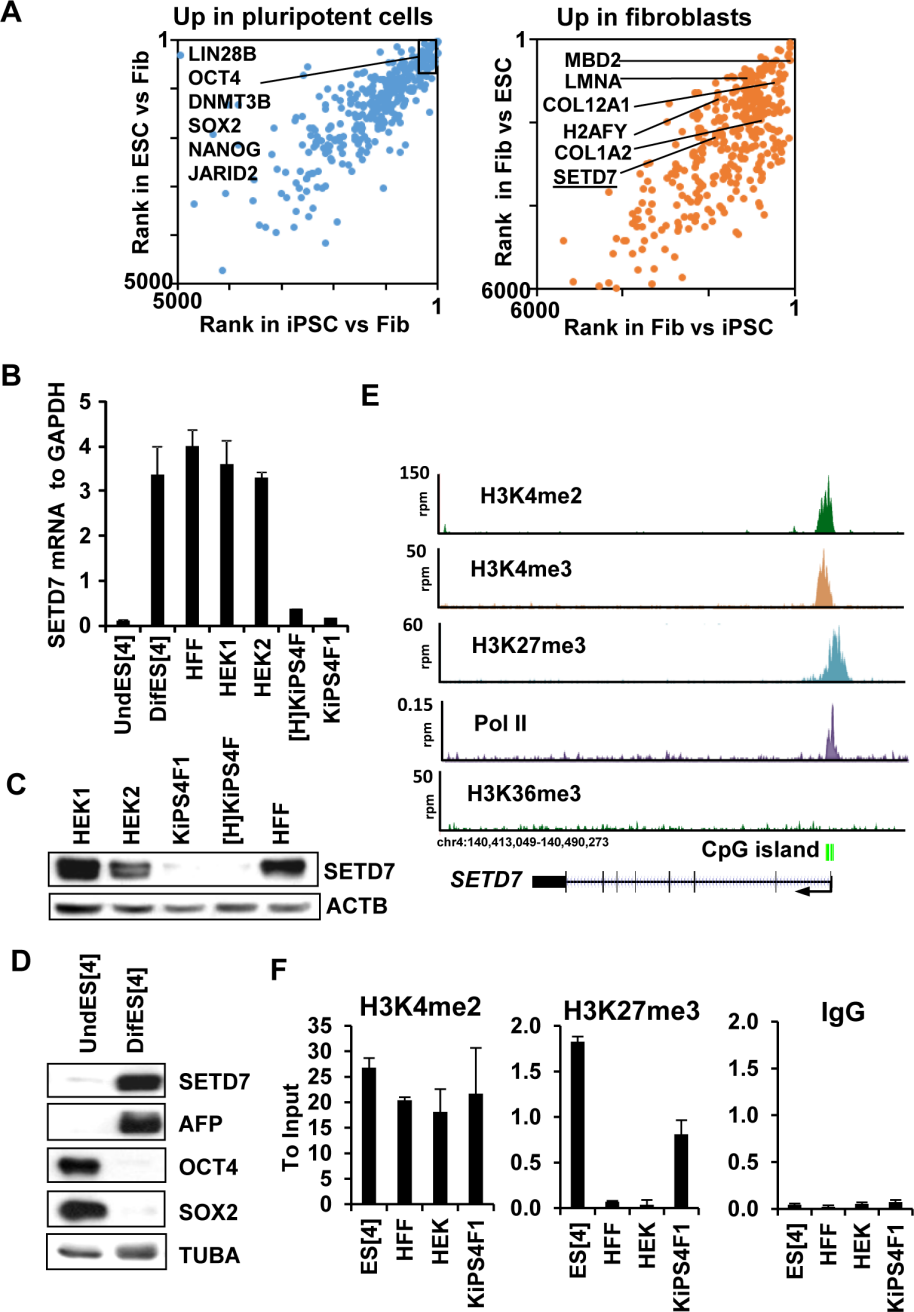
SETD7 is expressed at very low levels in pluripotent human cells and induced during differentiation. (A) Average rank of the top 700 most differentially expressed genes between pluripotent (iPSCs or ESCs) and fibroblasts, including those upregulated in pluripotent cells (left panel) and upregulated in fibroblasts (right panel). (B) *SETD7* mRNA levels in human ESCs grown under self-renewal conditions (UndES[4]), *in vitro* differentiated human ESCs (DifES[4]), human fibroblasts (HFF), two lines of human keratinocytes (HEK1 and HEK2) and two lines of iPSCs generated from keratinocytes ([H]KiPS4F and KiPS4F1). Mean and standard deviation of three technical replicates is shown. Induction of SETD7 mRNA levels during ES[4] differentiation was confirmed in more than four independent differentiation experiments. (C) Western blot showing SETD7 protein levels in pluripotent and somatic cells. Loading control beta actin (ACTB) is also shown. (D) Western blot showing protein levels of SETD7, AFP, OCT4 and SOX2 in under self-renewing conditions and *in vitro* differentiated human ESCs. Loading control alpha tubulin (TUBA) is also shown. One representative experiment out of three is shown. (E) Genomic visualization of the levels of H3K72me3, H3K4me3, H3K4me2, H3K36me3 and RNA polymerase II (Pol II) in the human embryonic stem cell line H1 around the *SETD7* gene according to ENCODE. A non-methylated CpG island is depicted in green. (F) Levels of H3K4me2 and H3K27me3 at *SETD7* gene promoter region (27 bp upstream of the transcription start site) in pluripotent and somatic cells determined by chromatin immunoprecipitation (ChIP) and ploted relative to the input. IgGs wer used as negative control. Bars show the mean and standard deviation of three independent immunoprecipitations.

In order to identity factors that play critical roles in the regulation of cell identity, we previously compiled publically available genome-wide expression data from 15 human iPSC lines and their fibroblasts of origin, and 12 human ESC lines [6]. For each set of data candidates were ranked according to their differential expression between pluripotent and somatic cells and top 1000 ranked genes were selected based on the average rank from all comparisons [6]. The top ranked differentially expressed genes in both iPSCs and ESCs compared to fibroblasts are shown in Fig 1A (See also S1 Table). As expected, pluripotency-related factors like OCT4, SOX2 and NANOG as well as previously reported chromatin-related factors DNMT3B and JARID2 were highly ranked as upregulated in pluripotent cells, while fibroblasts specific genes COL12A1 and COL1A2 were ranked as highly expressed in fibroblasts. Additionally, chromatin related factors such as MBD2, LMNA and H2AFY, previously described to be preferentially expressed in somatic cells versus pluripotent cells [7,40,41] appeared as highly expressed in fibroblast (Fig 1A). This result suggests that our method can robustly identify genes that are differentially expressed between somatic and pluripotent cells.

Among all candidates, we noticed that the protein lysine methyltransferase SETD7 was highly ranked for upregulated expression in fibroblasts versus pluripotent cells (Fig 1A). Quantification of mRNA levels by qPCR confirmed that *SETD7* was clearly up-regulated in keratinocytes and fibroblasts compared to iPSCs derived from keratinocytes (KiPSCs) (Fig 1B). Differences in expression were also confirmed at the protein level (Fig 1C). Also, SETD7 was expressed at higher levels in *in vitro* differentiated ESCs compared to ESCs maintained in undifferentiated conditions both at the mRNA (Fig 1B) and protein level (Fig 1D). Analysis of ENCODE data (Fig 1E) suggests that the promoter of SETD7 is marked with both H3K4me2/3 and H3K27me3 in the human embryonic stem cell line H1(Fig 1E), a combination of marks known as bivalent domains [42,43]. Bivalent domains have been suggested to keep differentiation genes poised for activation in pluripotent cells and to resolve into only H3K4me2/3 in genes that become activated during differentiation [42]. Additionally, RNA polymerase II (Pol II) was located that the transcriptional start site of *SETD7* but no elongation mark (H3K36me3) was detected at the gene body suggesting that Pol II is poised at the SETD7 promoter and ready for activation in human embryonic stem cells. Last, the presence of a non-methylated CpG island is in agreement with the Polycomb complex recruitment to the SETD7 promoter [44,45].

To confirm the presence of bivalent marks and resolution of these marks in somatic cells we performed chromatin immunoprecipitation (ChIP) in the different cell lines. In agreement with the differential expression of *SETD7* in pluripotent and somatic cells, the regulatory regions of *SETD7* were found to be marked only with H3K4me2 in somatic cells but co-marked with H3K27me3 in pluripotent cells (Fig 1F). The presence of this bivalency in pluripotent cells suggests that *SETD7* is silent in pluripotent cells but poised for activation during differentiation, similar to other critical developmental regulators [42,43].

### SETD7 plays a role in the differentiation of human ESCs

**Fig 2:**
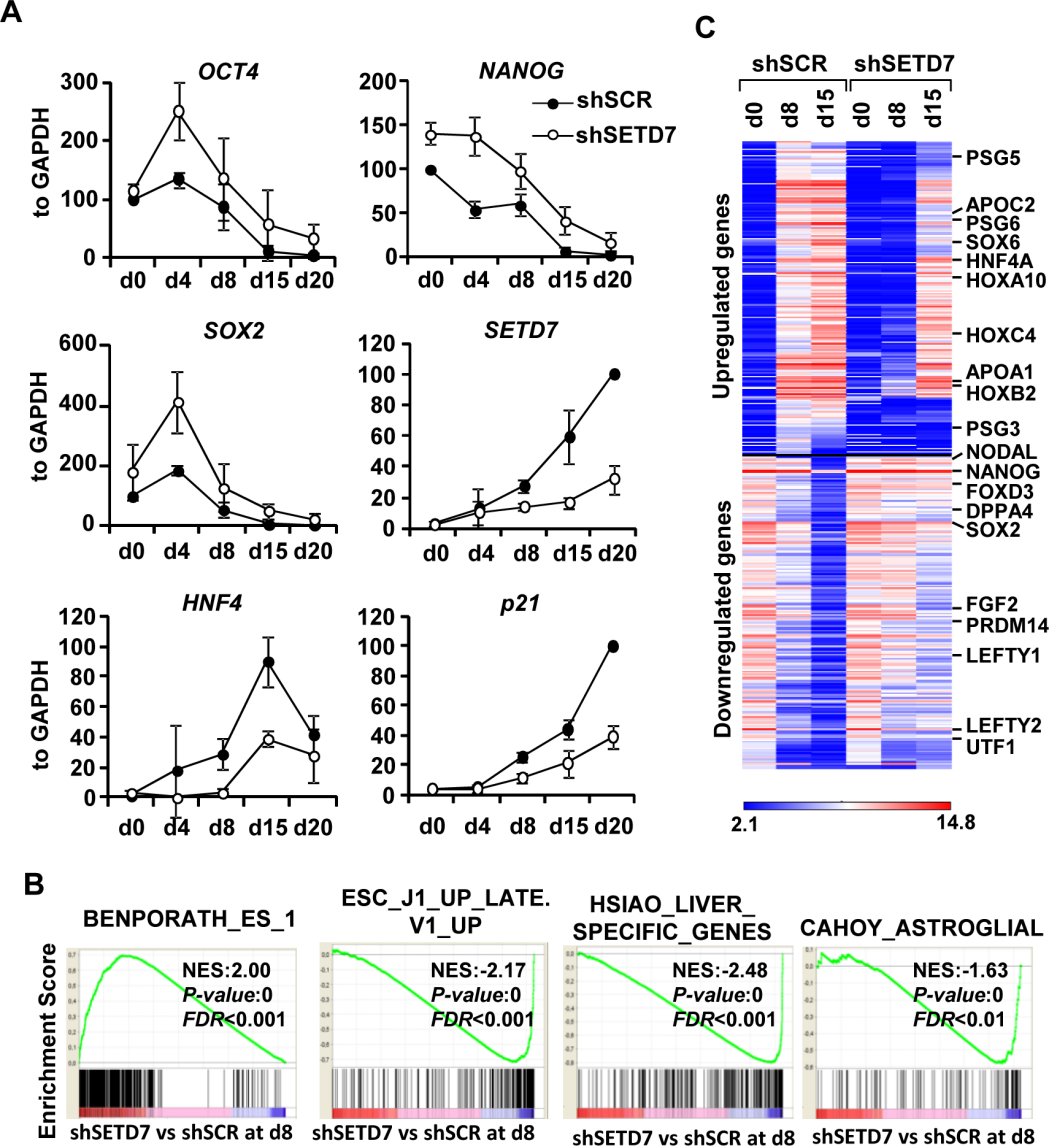
The SETD7 knock-down causes a delay in differentiation. (A) mRNA levels of pluripotency factors (*OCT4*, *SOX2*, *NANOG*), differentiation genes (*HNF4* and *p21*) and *SETD7* normalized to *GAPDH* at different days during the *in vitro* differentiation of ES[4] transduced with a non target shRNA (shSCR) and a shRNA (sh28) that targets SETD7 (shSETD7). For pluripotency factors levels were plotted relative to d0 and for differentiation factors levels were plotted relative to day of maximum expression in the shSCR. Mean and standard deviation from three independent differentiation experiments is shown. (B) Gene set enrichment analysis (GSEA) of the global changes in gene expression found between shSETD7 and shSCR at day 8 of differentiation. Enrichment profile, normalized enrichment score (NES), p-value and false discovery rate (FDR) are shown for the significantly enriched gene sets from the Molecular Signatures Database BENPORATH_ES_1 (genes overexpressed in human embryonic stem cells according to 5 or more out of 20 profiling studies), HSIAO_LIVER_SPECIFIC_GENES (Liver selective genes), ESC_J1_UP_LATE.V1_UP (Genes up-regulated during late stages of differentiation of embryoid bodies from J1 embryonic stem cells) and CAHOY_ASTROGLIAL (Genes up-regulated in astroglia cells). (C) Heatmap of the expression of the 400 genes most differentially regulated by shSETD7 KD compared to shSCR and that are upregulated or downregulated more than 16 fold during differentiation determined by microarray analysis.

To test if SETD7 has a role in ESCs differentiation, we proceed to knock down the expression of SETD7 using lentiviral vectors that express shRNAs against SETD7. Five different shRNAs from SIGMA (MISSION) were tested in 293T and ESCs. Two particular shRNAs (sh28 and sh29) showed a significant reduction of SETD7 mRNA and protein levels both in somatic cells as well as in in vitro differentiated ESCs (S1A, S1B and S1C Fig). ESCs knocked down for SETD7 by means of sh28 expression (shSETD7) were differentiated *in vitro* and levels of pluripotency and differentiation-related genes were quantified at different days using qPCR. Fig 2A shows that the silencing of pluripotency-related genes *OCT4*, *NANOG* and *SOX2* is delayed in SETD7 knock-down cells compared to cells infected with a control shRNA (shSCR), as well as the induction of differentiation genes HNF4 and p21. Microarray analysis was performed at different days during differentiation. Fig 2B shows that genes that are upregulated in the shSETD7 condition compared to the shSCR at day 8 of differentiation are enriched in pluripotency genes while genes downregulated are mainly differentiation genes, including genes expressed in liver and astroglia. These results suggest that the SETD7 knock down is affecting both the silencing of pluripotency genes and the induction of liver specific genes during differentiation. Differentially expressed genes (Fig 2C) included genes of the pluripotency network such as OCT4, SOX2, NANOG, UTF, PRDM14 and TERT among others (S2A Fig) and differentiation genes including HOX genes, apolipoproteins, pregnancy specific glycoproteins (expressed in trophectoderm), genes involved in liver (HNF4A, HMGCS2), muscle (TNNI1), blood (MEIS1, SOX6) and brain differentiation (CBLN2 and KLK6) (S3A Fig). Most differentially expressed genes induced or silenced during differentiation were not significantly affected by the SETD7 knock down in undifferentiated cells (S2C and S3C Figs) in accordance with the low levels of expression of SETD7 in these cells.

**Fig 3:**
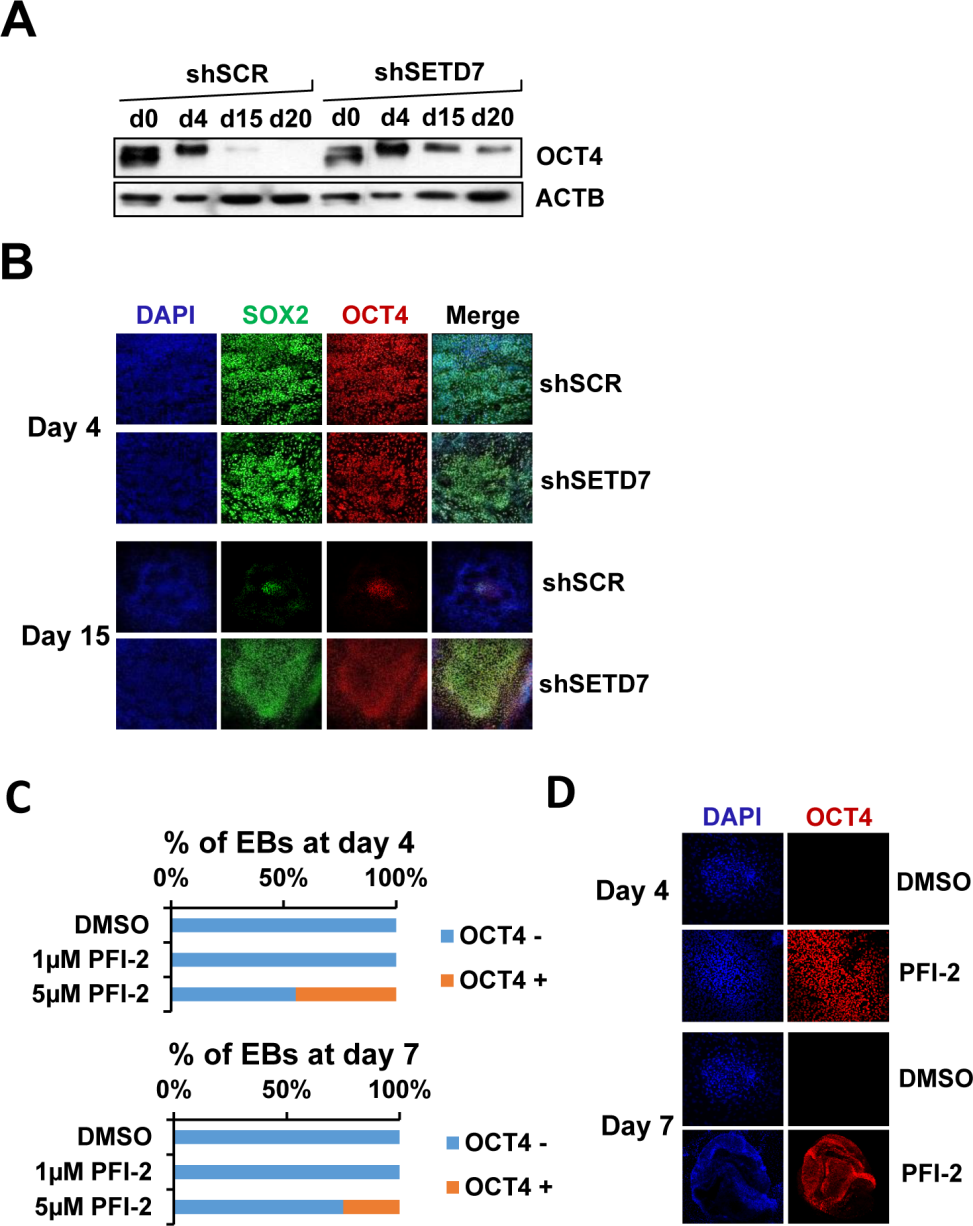
The SETD7 knock-down causes defects in the silencing of pluripotency genes. (A) Western blot showing the levels of OCT4 at different days during the in vitro differentiation of ES[4] transduced with a non target shRNA (shSCR) and a shRNA that targets SETD7 (shSETD7) (B) Immunolocalization of SOX2 (green) and OCT4 (red) expression at day 4 and day 15 of in vitro differentiation of ES[4] transduced with a non target shRNA (shSCR) and a shRNA that targets SETD7 (shSETD7) (C) Quantification of the percentage of embryoid bodies negative or positive for OCT4 staining at day 4 and day 7 of differentiation of cells treated with vehicle (DMSO), 1μM or 5μM PFI-2. (D) Immunolocalization of OCT4 (red) in embryoid bodies at day 4 and day 7 of in vitro differentiation of ES[4] treated with vehicle (DMSO) or 5μM PFI-2.

Delayed silencing of the pluripotency-related gene OCT4 was further confirmed at the protein level by western blot (Fig 3A) and immunohistochemistry (Fig 3B). Additionally, treatment of cells during differentiation with the SETD7 inhibitor PFI-2 [46] at 5μM caused the sustained expression of OCT4 in a percentage of embryoid bodies compared to the vehicle (DMSO) treatment (Fig 3C and 3D).

**Fig 4:**
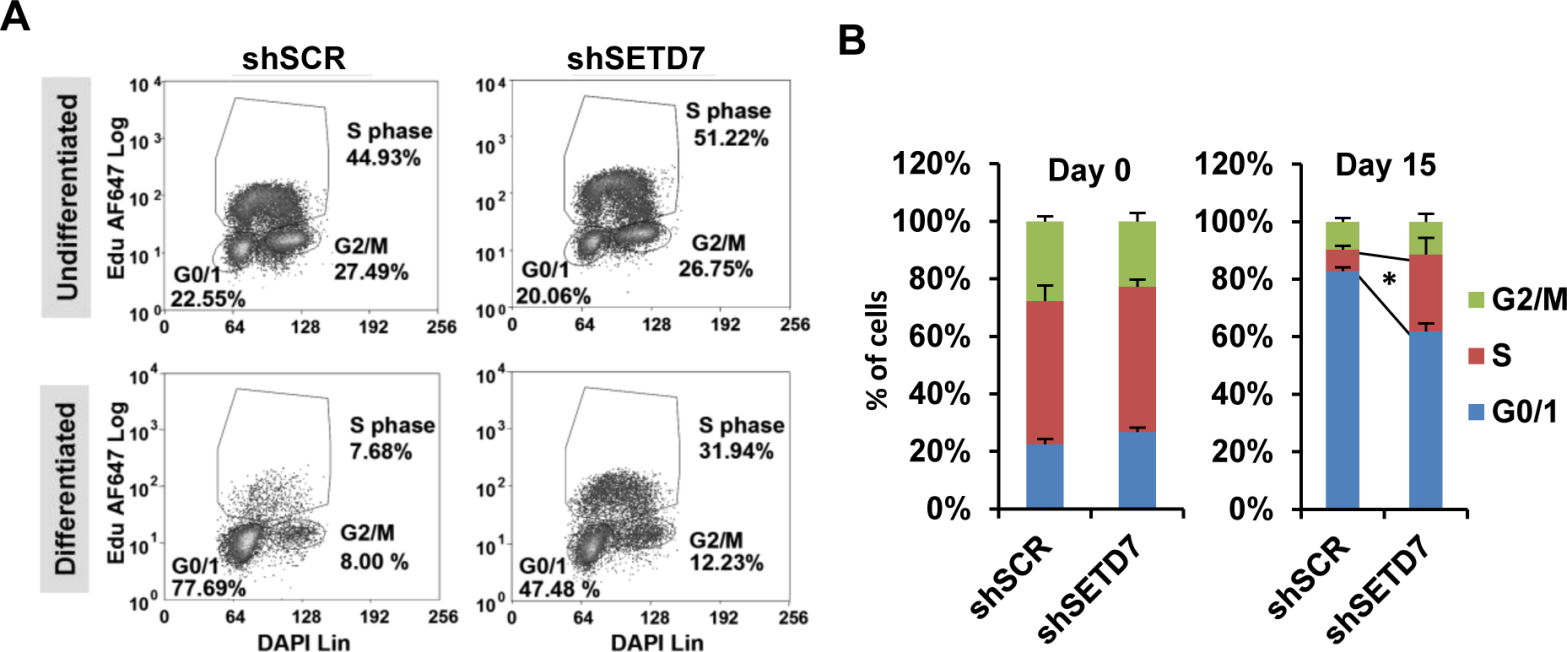
The SETD7 knock-down affects the cell cycle profile of differentiating cells. (A) Cell cycle profile of undifferentiated and after 15 days of *in vitro* differentiation of ES[4] transduced with a non target shRNA (shSCR) and a shRNA that targets SETD7 (shSETD7). (B) Mean of the percentage of cells in each phase of the cell cycle in three independent differentiation experiments. *Differences in the percentage of cells in S-phase between shSETD7 and shSCR in differentiated cells was found significant at a p-value<0.05.

To further confirm the involvement of SETD7 in differentiation we evaluated the cell cycle distribution of undifferentiated and differentiated ESCs control and knocked down for SETD7. As expected control cells showed a dramatic decrease of cells in S-phase upon differentiation (Fig 4A and 4B), while cells knocked down for SETD7 showed a significant number of cells in S phase upon differentiation. These results are consistent with the delays in differentiation found in SETD7 knock-down cells at the level of gene expression.

### SETD7 binds and methylates histone H1

Accumulating evidence suggests that SETD7 might mediate its physiological effects through the methylation of proteins different than histone H3 [10,12,25,34,47]. Additionally, the defective silencing of pluripotency-related genes observed during the differentiation of SETD7 knock-down ESCs can hardly be explained by defects on H3K4me1, since this epigenetic mark is involved in gene activation. Therefore, we set up to explore other potential methylation targets that might mediate the physiological role of SETD7 during differentiation.

**Fig 5:**
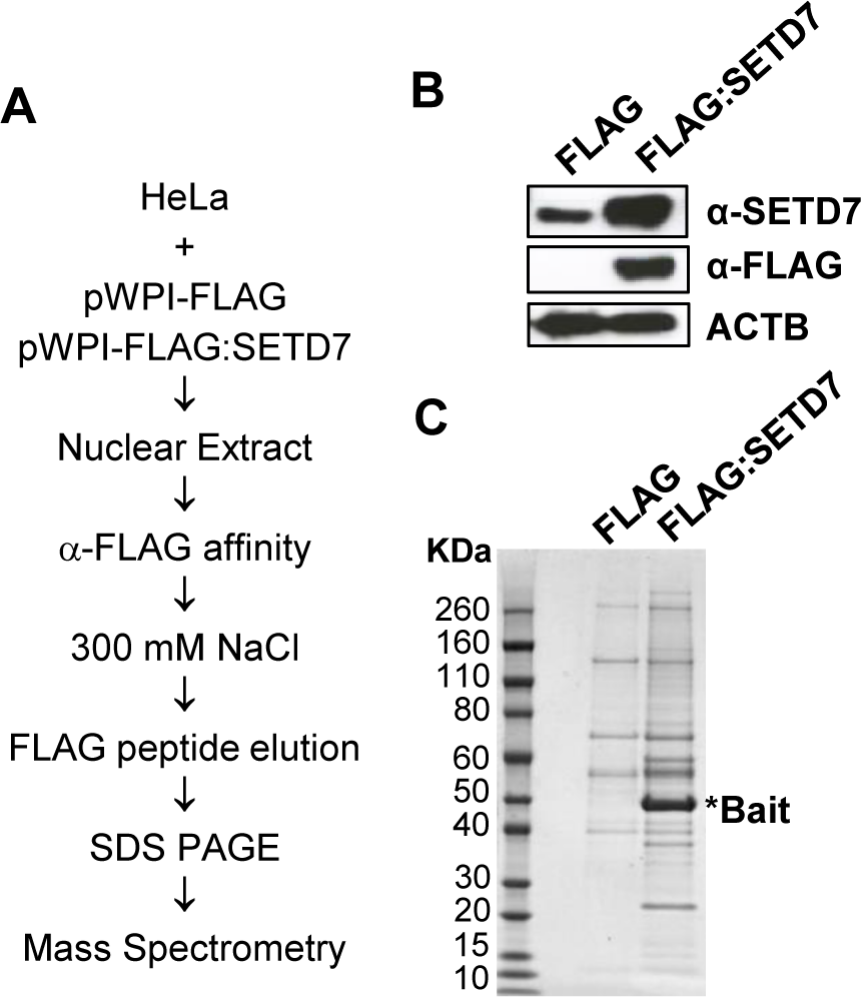
Analysis of SETD7 interacting proteins. (A) Strategy for the purification of interacting proteins. (B) Levels of endogenous and overexpressed SETD7 in infected HeLa cells infected with pWPI-FLAG (FLAG) or pWPI-FLAG:SETD7 (FLAG:SETD7) determined by western blot. (C) Coomassie blue staining of the immunoprecipitated proteins. Molecular weight markers, anti FLAG-immunoprecipitated proteins from cells transduced with empty vector and cells transduced with FLAG:SETD7 expressing vector are shown.

Since SETD7 is expressed mostly in differentiated cells we hypothesized that the methylation target through which SETD7 might be influencing the differentiation of pluripotent cells is likely to be expressed in differentiated cells. In order to identify potential targets of SETD7 in differentiated cells we analyzed SETD7 interacting proteins by mass spectrometry in HeLa cells (Fig 5A). For that, FLAG tagged SETD7 was overexpressed in HeLa cells (Fig 5B). Overexpression did not change localization of SETD7 in HeLa cells (S1D Fig). Soluble nuclear extracts were prepared and FLAG:SETD7 was immunoprecipitated using anti FLAG antibodies. Two independent immunoprecipitations were carried out in which the immunoprecipitated material was resolved in a SDS-polyacrylamide gel and subjected to in gel digestion (Fig 4C) or digested in solution for further analysis by mass spectrometry. Table 1 shows the number of spectral counts for proteins that were significantly enriched in the anti-FLAG:SETD7 immunoprecipitation compared to the control (cells infected with pWPI:FLAG). Interacting proteins were mainly enriched in RNA splicing (p-value: 2.3 E-9) and gene expression (p-value: 6.2E-4) categories.

**Table 1:** SETD7 interacting proteins identified by mass spectrometry.

	In gel		In Solution		
	FLAG	F:SETD7	FLAG	F:SETD7	
**SETD7**	9	123	0	255	**Bait**
PHF5A	0	2	0	1	
HNRNPF	0	1	0	11	
PTBP1	0	1	1	5	
SRRM2	0	1	1	7	
SNRPB	0	6	0	1	**Splicing/RNA procesing**
SF3B4	0	2	0	1	
SFRS1	0	2	0	2	
SFRS3	0	5	0	2	
TRA2B	0	1	0	2	
FBL	2	12	0	3	
LUC7L2	0	2	0	4	
SRP14	0	10	0	2	
CAPRIN1	0	1	0	1	
HIST1H1C	0	7	0	2	
GATAD2B	0	2	0	1	
LMNA	0	1	4	30	
DEK	0	1	0	1	**Transcription/Nuclear organization**
RUVBL1	0	3	0	1	
PRKDC	5	114	8	21	
RIF1	0	1	1	4	
PCNA	0	5	0	1	
EEF1D	0	12	0	8	
PSMC1	0	4	0	1	**Others**
PHGDH	0	3	1	2	
PFKFB2	3	20	0	8	

The table shows the number of total peptide spectra matched to the indicated proteins per condition.

Among interacting proteins we noticed the presence of linker histone H1.2 (HIST1H1C) that has prominent roles in the regulation of chromatin structure and gene expression. The interaction of SETD7 with H1.2 suggested that linker histone H1 could be a target for SETD7-mediated methylation. In accordance, we found that the treatment of HeLa cells with 5μM PFI-2 reduced the levels of methylation of the histone fraction (including H1 variants) compared to cells treated with vehicle (S4 Fig).

**Fig 6:**
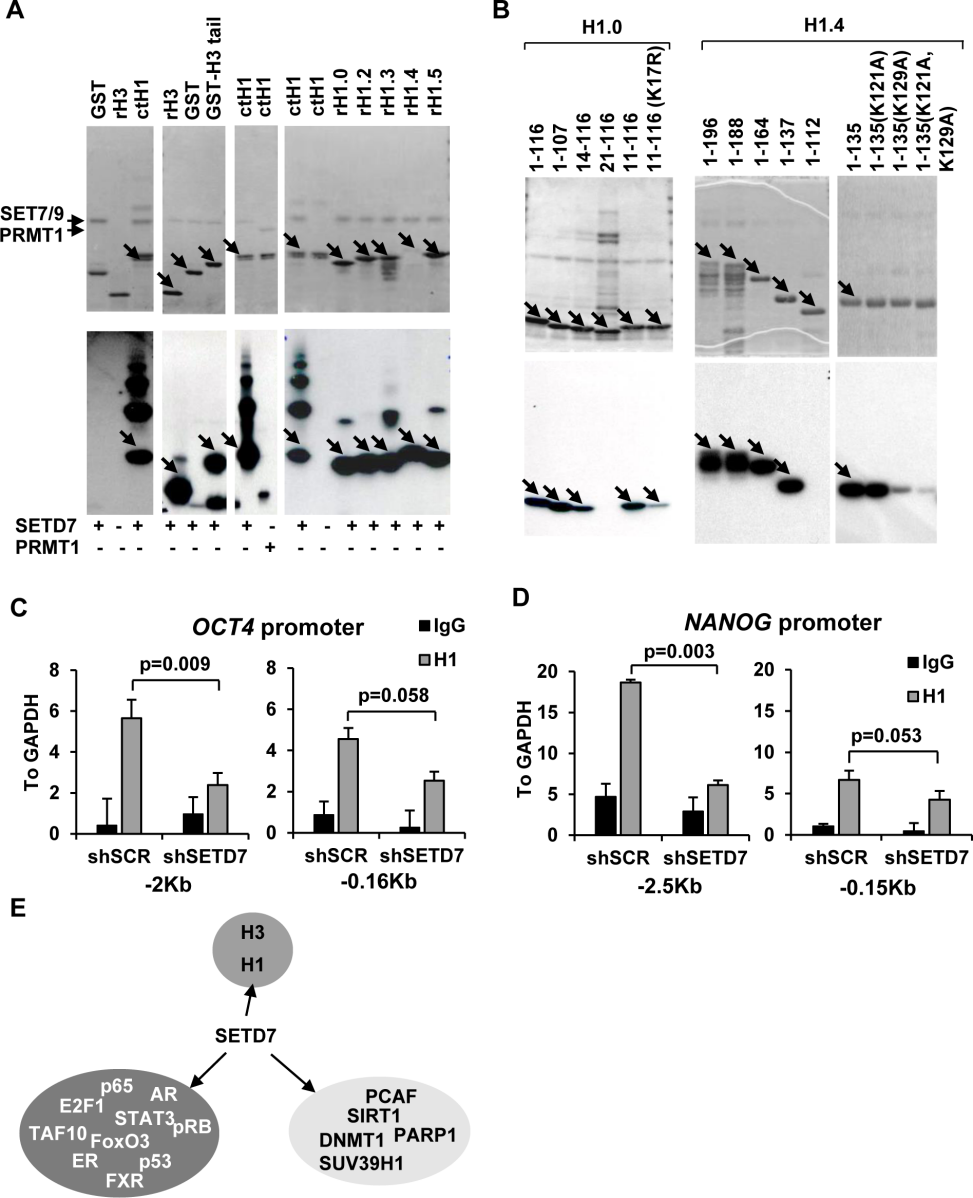
SETD7 methylates linker histone H1 and affects its recruitment to chromatin during differentiation. (A) *In vitro* methylation reactions showing the activity of SETD7 and PRMT1 on histones H3 and H1. Coomassie blue staining of SDS-PAGE gels (upper panel) and autoradiography (lower panel) of four different gels are shown. (B) *In vitro* methylation reactions mapping the methylation sites of linker histones H1.0 and H1.4 by SETD7. Coomassie blue staining of SDS-PAGE gels (upper panel) and autoradiography (lower panel) of three different gels are shown. Arrows show the expected molecular weight of the recombinant proteins. (C) Chromatin immunoprecipitation showing the recruitment of total H1 to two regions of the OCT4 promoter at 15 days of *in vitro* differentiation of ES[4] transduced with a non target shRNA (shSCR) and a shRNA that targets SETD7 (shSETD7). Positions indicate Kb from the transcription start site. Levels were normalized to the input and plotted relative to a negative control region in the *GAPDH* gene promoter. Mean and standard deviation of three independent differentiation experiments is shown. (D) As in C but testing two regions of the NANOG promoter (E) Reported SETD7 methylation targets.

To confirm the methylation of H1 variants by SETD7 we conducted *in vitro* methylation assays. SETD7 was able to methylate full length recombinant histone H3 and histone H3 tail (Fig 6A), as previously described. SETD7 was also able to methylate a preparation of linker histones from calf thymus, while the arginine methyltransferase PRMT1 was unable to do so. We next confirmed that SETD7 was able to methylate all the tested recombinant H1 histone variants (Fig 6A, right panel). Among them, two particular variants were selected for follow up; H1.4 because it showed a high methylation activity despite the low levels of protein assayed and H1.0 because it has been previously suggested to play a role in human ESCs differentiation [48]. *In vitro* methylation assays using deletions and point mutations in recombinant H1.0 revealed that the main methylation site was located at K17 in the N-terminal domain (Fig 6B, left panel) which was confirmed by mass spectrometry (Table 2). Histone H1.4 was found to be methylated at K129 in the C-terminal domain since mutations at this site dramatically affected the levels of methylation (Fig 6B, right panel). Mass spectrometry data (Table 2) confirmed this methylation site and revealed the presence of an additional methylation site in the N-terminal domain at K34.

**Table 2:** Residues modified by SETD7 in H1.0 and H1.4 found by mass spectrometry.

Histone	Methylated K	# modified peptides
**H1.0**	**K12**	**4**
	**K14**	**3**
	**K17**	**42**
	**K20**	**13**
	**K17&K20**	**10**
	**K21**	**5**
	**K20&K21**	**1**
	**K27**	**1**
	**K111**	**1**
**H1.4**	**K34**	**21**
	**K127**	**9**
	**K129**	**21**
	**K130**	**3**

The table shows the number of modified peptides containing the indicated modifications after the analysis of in vitro methylated H1.0 (construct 1–116 aa) or H1.4 (construct 1–135) by mass spectrometry.

### Methylation of H1.4 by SETD7 causes conformational changes

In order to understand the biological function of H1 methylation by SETD7 we tested if methylation of H1.4 could be causing conformational changes. Therefore, we analyzed the effect of methylation by SETD7 in the secondary structure of H1.4 in solution and bound to DNA by FTIR. In aqueous solution the methylated protein (4 methyl groups incorporated on average, see S5A Fig) showed no significant changes when compared to unmethylated H1.4 (S5B and S5C Fig). However, differences in the amount of secondary structure were observed upon DNA binding. In the complexes the percentages of α-helix increased from 20% to 27% in the unmethylated protein, while those of β-structure only had a slight increase from 22% to 26% in the presence of DNA. The opposite was true for the complexes with methylated H1.4 where the greatest increase was in β-structure from 25% to 33%, while the amount of α-helix remained almost unchanged (S5B and S5C Fig). In conclusion, H1.4 methylated by SETD7, upon binding to DNA, tended to form less α-helix but more β-structure than unmethylated H1.4.

### SETD7 regulates the incorporation of histone H1 during differentiation

We have previously reported that histone H1 is incorporated into the regulatory regions of pluripotency genes to mediate their silencing during the differentiation of human ESCs [48]. Therefore we set up to quantify if the silencing of SETD7 expression could be affecting the incorporation of linker H1 to these regions during differentiation. Chromatin immunoprecipitation experiments confirmed that H1 incorporation to the pluripotency-related genes *OCT4* and *NANOG* during differentiation is lower in SETD7 knock-down cells, suggesting that H1 methylation by SETD7 could contribute to the recruitment of H1 to pluripotency genes during differentiation and their silencing (Fig 6C). Importantly, the expression levels of H1 variants were not significantly affected by the shSETD7 during differentiation (S3C Fig).

## Discussion

Growing evidence suggests that not only classical transcription factors are induced during differentiation to orchestrate the expression of tissue specific programs. Important chromatin regulators are also developmentally regulated and contribute to regulate the expression of the appropriate set of genes in differentiated cells. Here, we show that SETD7 is strongly upregulated during the differentiation of human pluripotent cells and differentially expressed between iPSCs and somatic cells. Furthermore, although the role of bivalent domains in repressing developmental genes in ESCs remains controversial [49], the fact that SETD7 is marked with bivalent domains in pluripotent cells further suggests that it is an early important developmental regulator.

Our results raise the possibility that methylation of histone H1 by SETD7 could be involved in the regulation of ESCs differentiation. All tested H1 variants were found to be methylated by SETD7 *in vitro*, including H1.2 that was identified as a SETD7 interacting protein in the immunoprecipitation experiments. Further analysis of methylation sites revealed that histone H1.4 was found to be methylated *in vitro* by SETD7 at K129 in the C-terminal domain and at K34 in the N-terminal domain. Our report is consistent with a recent paper that describes methylation of H1.4 at several lysines on the C-terminal domain [50]. Moreover, our work shows that K129 is the key residue for SETD7 driven methylation, since its mutation greatly reduces methylation levels *in vitro*. Despite this fact, mass spectrometry data showed prominent levels of K34 methylation when analyzing the wild type recombinant H1.4. Interestingly, H1.4 K34 has been found to be acetylated and methylated *in vivo* [51,52]. Acetylation of K34 increases H1.4 mobility and it is particularly prominent in pluripotent cells [51], likely contributing to the hyperdynamic binding of linker histones to chromatin in these cells [53]. We hypothesize that the induction of SETD7 during differentiation will lead to methylation of H1.4 at K34 and therefore reduce the levels of acetylation by competition, contributing to the establishment of the proper heterochromatin patterns during differentiation. Additionally, K34 of H1.4 is conserved in H1.2 and H1.3 variants which have been previously found to be methylated, acetylated and formylated at this residue in mouse tissues [52]. These data suggests that the target sites of SETD7 methylation in H1 histones might be subject of intense regulation by posttranscriptional modifications.

Upon methylation by SETD7, differences in the percentages of secondary structure of H1.4 when bound to DNA were observed, suggesting DNA-dependent conformational changes in H1.4. Those changes are presumably due to a different folding of H1 terminal domains determined by the incorporation of methyl groups. Conformational changes associated with posttranslational modifications and characterized by an increase in β-structure at the C-terminal domain were previously described for H1 bound to DNA and in chromatin [54,55]. Recognition of methylated positions in H3 by several chromatin readers, including HP1, are mediated by β-sheet protein−protein interactions [56,57], so conformational changes induced by H1 methylation could affect interactions with consequences on chromatin organization or compaction. Alternatively, H1 methylation by SETD7 may influence its turnover or exchange.

We have previously reported a role for histone H1 in the silencing of pluripotency-related genes during the differentiation of human embryonic stem cells [48]. Differentiation entails changes in total H1 content, as well as changes in the relative levels of H1 variants. Most prominent change is the induction of H1.0 variant and recruitment to pluripotency-related genes to facilitate their silencing. Here, we propose that the induction of SETD7 expression might be also contributing to the recruitment of H1 to those genes during differentiation.

Due to the complexity of the differentiation process and the multiple targets that have been proposed for SETD7 (Fig 5E) a more systematic approach will be needed in the future to dissect the specific effects of SETD7 on H1 biology. For example, raising specific antibodies against methylated H1 to investigate how and where methylated H1 is incorporated into chromatin during differentiation would be of interest. Additionally, performing mass spectrometry to compare the modification patterns of H1 in differentiated and undifferentiated cells could be appropriate. Moreover, our co-immunoprecipitation analysis suggest that additional proteins might interact and be potentially methylated by SETD7 or regulate SETD7 activity. We have shown here the common interactors found in two independent immunoprecipitations. However, since co-immunoprecipitation is not exempt from artifacts and false positives, additional experiments will be needed to carefully verify the specificity of these interactors.

Since SETD7 is able to methylate multiple targets one might anticipate widespread effects upon differentiation. Accordingly, a report published during the preparation of this manuscript suggests that SETD7 can contribute to the differentiation of ESCs by methylating and regulating the stability of the pluripotency factor SOX2 [58]. Eventually, methylation of other targets such as YAP or LIN28A could be potentially involved in the effects of SETD7 in differentiation [59–61]. Although most of the current effort in the field has been dedicated to the characterization of changes in the transcriptome during differentiation, regulation of protein translation is also emerging as a key regulatory mechanism [62]. Additionally, we suggest that differential expression of protein modifying enzymes could be contributing to the establishment of posttranslational modifications that play a key role in differentiation. Therefore, a closer look at the proteome and its modifications appears critical to fully understand the molecular events involved in differentiation.

## Conclusions

We show that SETD7 is expressed at very low levels in human ESCs and induced during differentiation. Knock-down of SETD7 causes differentiation defects in human embryonic stem cells. SETD7 methylates linker histone H1 in vitro causing conformational changes in H1. These effects correlate with a decrease in the recruitment of H1 to the pluripotency genes *OCT4* and *NANOG* during differentiation in SETD7 knocked down cells, which might affect the proper silencing of these genes during differentiation.  

## Supporting Information

S1 Fig Knock down efficiency of different shRNAs against SETD7. (A) Levels of *SETD7* mRNA normalized to *GAPDH* in undifferentiated ES[4] (UndES[4]), *in vitro* differentiated ES[4] for 15 days (DifES[4]) and 293T cells transduced with a non target shRNA (shSCR) and 5 different shRNAs against SETD7 (shSETD7). (B) Western blot showing the protein levels of SETD7 in 293T cells transduced with a non target shRNA (shSCR) and 5 different shRNAs against SETD7 (shSETD7). (C) Western blot showing the protein levels of SETD7 in undifferentiated ES[4] (UndES[4]) and *in vitro* differentiated ES[4] for 15 days (DifES[4]) cells transduced with a non target shRNA (shSCR) and 5 different shRNAs against SETD7. (D) Subcellular distribution of SETD7 in Hela cells infected with pWPI-FLAG and pWPI-FLAG:SETD7. Cytoplasmic and nuclear fractions are shown. Immunoblot of transcription factor AP-2 shows the enrichment of nuclear proteins in the nuclear vs. cytoplasmic fractions.
(PDF)

S2 Fig Expression of genes downregulated during differentiation affected by the shSETD7. (A) mRNA levels of different pluripotency-related genes during differentiation in the shSCR (full lines) and the shSETD7 (dotted lines) cell lines. (B) Blox plot of the levels of expression of the upregulated genes depicted in Fig 2C and p-values of the differential expression between the indicated categories according to t-test (C) Levels of expression of housekeeping genes during differentiation.
(PDF)

S3 Fig Expression of genes upregulated during differentiation affected by the shSETD7. (A) mRNA levels of several differentiation genes during differentiation in the shSCR (full lines) and the shSETD7 (dotted lines) cell lines. (B) Blox plot of the levels of expression of the downregulated genes depicted in Fig 2C and p-values of the differential expression between the indicated categories according to t-test (C) Heatmap of the levels of expression of H1 variants during differentiation of the shSCR and the shSETD7 cell lines.
(PDF)

S4 Fig Effects of the PFI-2 and Adox inhibitors in the methylation of the histone fraction. HeLa cells were labeled with [methyl-3H]-L-methionine for 3 h in the presence of protein-synthesis inhibitors, and in the presence of vehicle or the SETD7 inhibitor PFI-2 (5 μM) or the general methyltransferase inhibitor AdOx (20 μM). Acid extraction of histone fraction was performed and proteins resolved by SDS-PAGE, visualized by Coomassie blue staining, followed by autoradiography. Western blot with anti H1.0 antibody is shown as loading control. (*) Corresponds to methylated H1 bands.
(PDF)

S5 Fig Effect of methylation by SETD7 in the seconday structure of H1.4 in solution and bound to DNA. (A) Effect of methylation by SETD7 in the seconday structure of H1.4 in solution and bound to DNA. A, Mass spectrometry spectra of in vitro methylated H1.4 with 4 methyl groups incorporated on average, compared to unmethylated protein. (B) Infrared spectroscopy results for the unmethylated and methylated proteins in solution and bound to DNA. (C) Amide I decomposition of the unmethylated and methylated H1.4 in solution and bound to DNA. The α-helix component is highlighted in orange and the β-structure component is highlighted in light blue. Infrared measurements were performed at a protein concentration of 5 mg/ml in 10mM Hepes pH 7.0, plus 140 mM NaCl as described in Experimental Procedures. The protein/DNA ratio (r) (w/w) was 0.7.
(PDF)

S1 Table Ranking of the top 700 most differentially expressed genes between pluripotent cells (iPSCs and ESCs) and fibroblast. (XLS)

S2 Table Expression (Log2) of the top 400 most differentially expressed genes between shSCR and shSETD7 induced (UP) or repressed (DOWN) during differentiation. (XLS)
