# Supplementary figures and images for: SETD7 Regulates the Differentiation of Human Embryonic Stem Cells
Source: PLoS One. 2016 Feb 18;11(2):e0149502. doi: 10.1371/journal.pone.0149502 (PMC4758617; doi:10.1371/journal.pone.0149502)

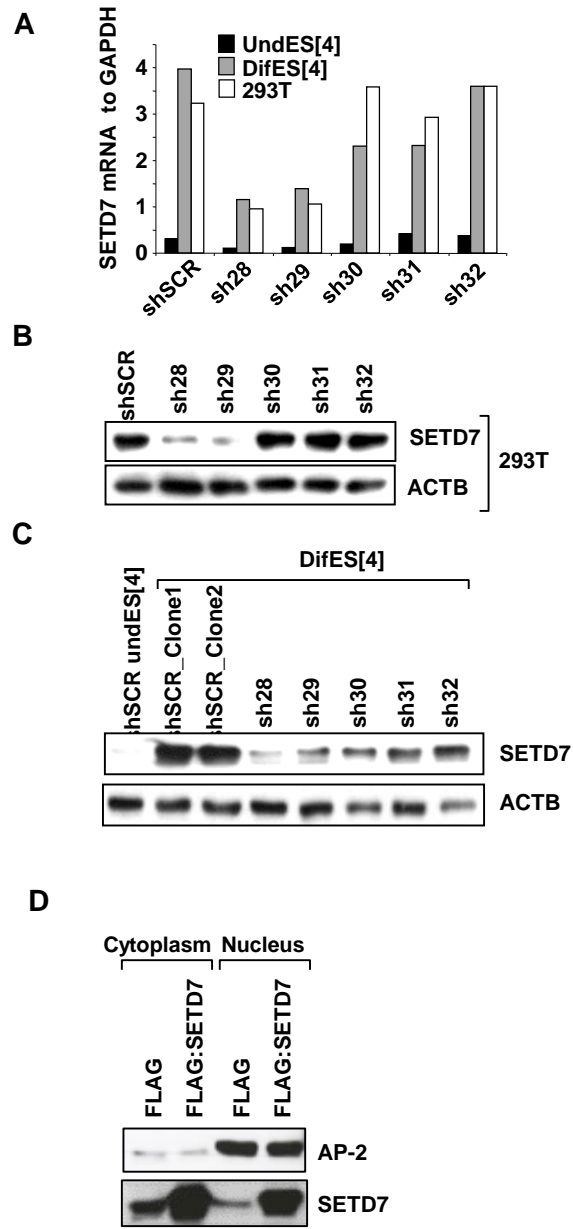

Supplement: S1 Fig — (A) Levels of SETD7 mRNA normalized to GAPDH in undifferentiated ES[4] (UndES[4]), in vitro differentiated ES[4] for 15 days (DifES[4]) and 293T cells transduced with a non target shRNA (shSCR) and 5 different shRNAs against SETD7 (shSETD7). (B) Western blot showing the protein levels of SETD7 in 293T cells transduced with a non target shRNA (shSCR) and 5 different shRNAs against SETD7 (shSETD7). (C) Western blot showing the protein levels of SETD7 in undifferentiated ES[4] (UndES[4]) and in vitro differentiated ES[4] for 15 days (DifES[4]) cells transduced with a non target shRNA (shSCR) and 5 different shRNAs against SETD7. (D) Subcellular distribution of SETD7 in Hela cells infected with pWPI-FLAG and pWPI-FLAG:SETD7. Cytoplasmic and nuclear fractions are shown. Immunoblot of transcription factor AP-2 shows the enrichment of nuclear proteins in the nuclear vs. cytoplasmic fractions. (PDF) [file pone.0149502.s001.pdf]

S2 Figure

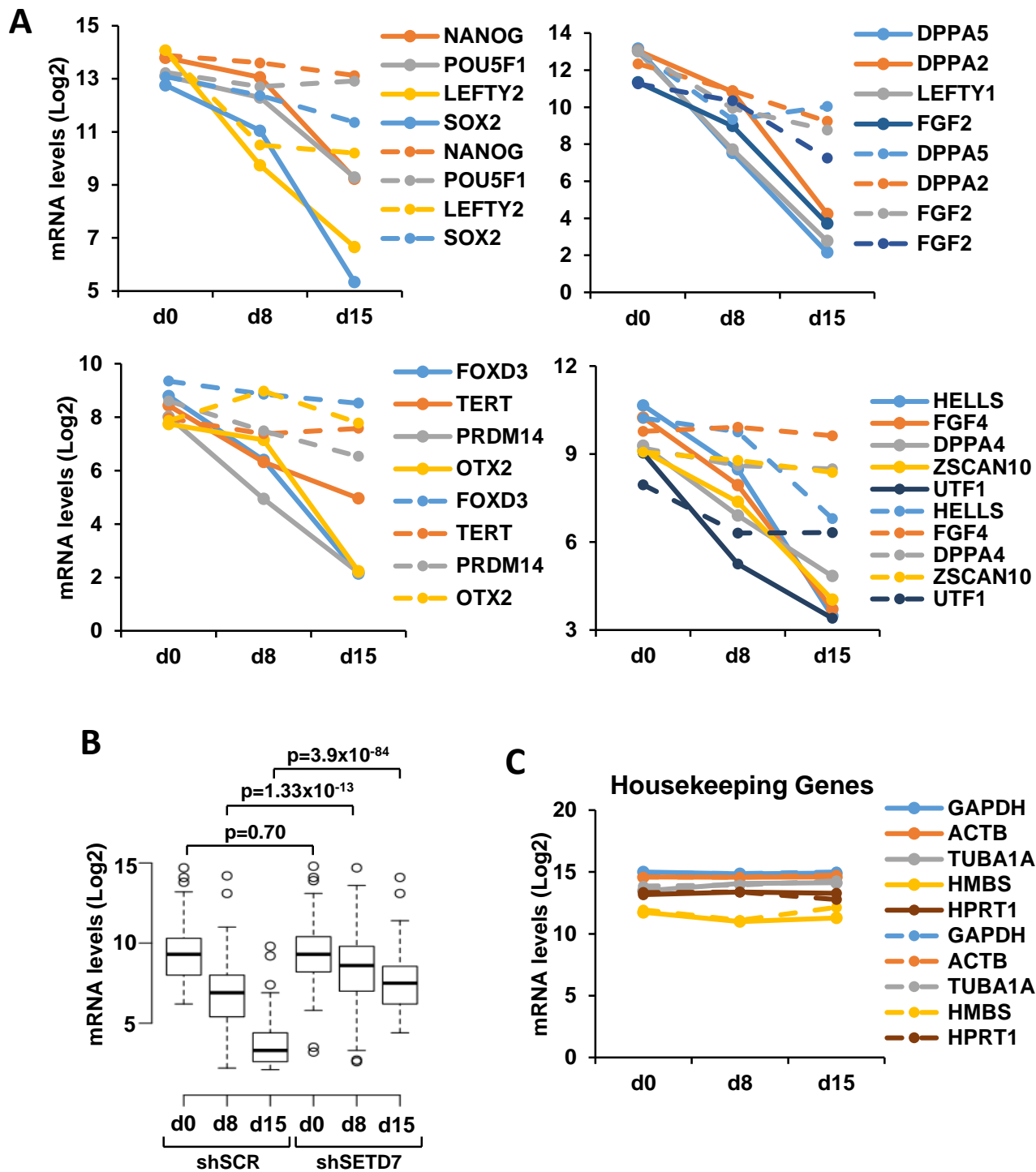

Supplement: S2 Fig — (A) mRNA levels of different pluripotency-related genes during differentiation in the shSCR (full lines) and the shSETD7 (dotted lines) cell lines. (B) Blox plot of the levels of expression of the upregulated genes depicted in Fig 2C and p-values of the differential expression between the indicated categories according to t-test (C) Levels of expression of housekeeping genes during differentiation. (PDF) [file pone.0149502.s002.pdf]

S3 Figure

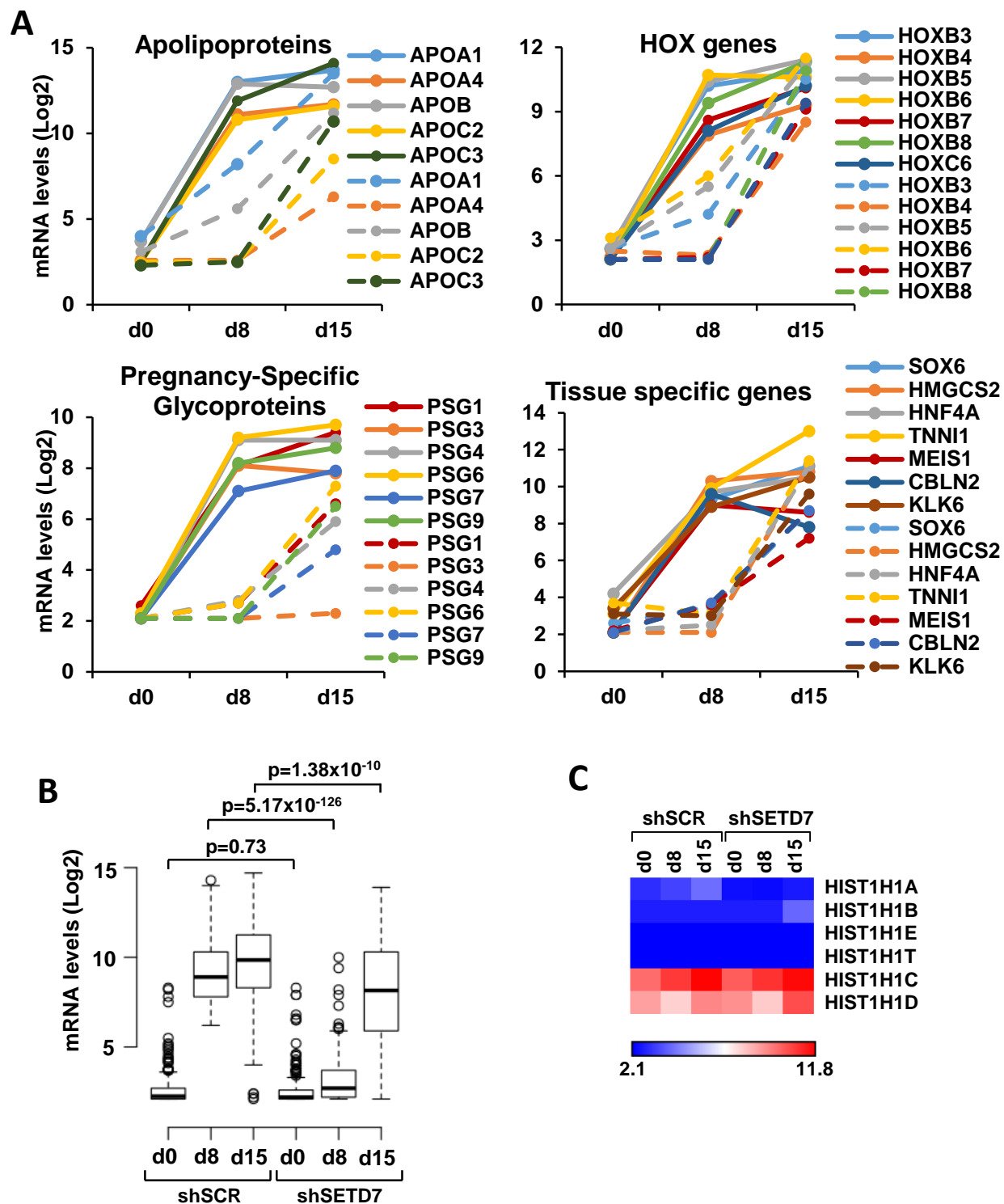

Supplement: S3 Fig — (A) mRNA levels of several differentiation genes during differentiation in the shSCR (full lines) and the shSETD7 (dotted lines) cell lines. (B) Blox plot of the levels of expression of the downregulated genes depicted in Fig 2C and p-values of the differential expression between the indicated categories according to t-test (C) Heatmap of the levels of expression of H1 variants during differentiation of the shSCR and the shSETD7 cell lines. (PDF) [file pone.0149502.s003.pdf]

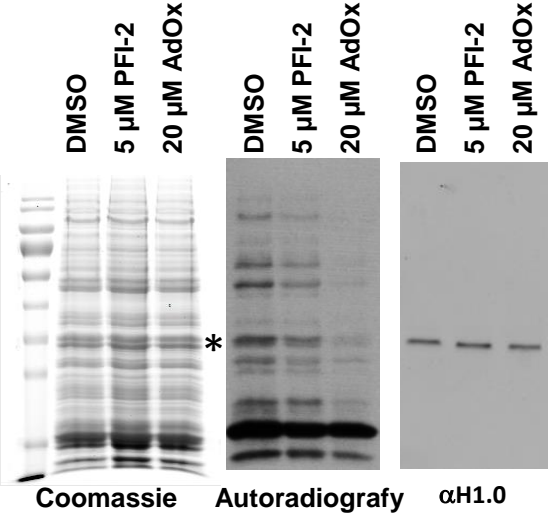

Supplement: S4 Fig — HeLa cells were labeled with [methyl-3H]-L-methionine for 3 h in the presence of protein-synthesis inhibitors, and in the presence of vehicle or the SETD7 inhibitor PFI-2 (5 μM) or the general methyltransferase inhibitor AdOx (20 μM). Acid extraction of histone fraction was performed and proteins resolved by SDS-PAGE, visualized by Coomassie blue staining, followed by autoradiography. Western blot with anti H1.0 antibody is shown as loading control. (*) Corresponds to methylated H1 bands. (PDF) [file pone.0149502.s004.pdf]
