# Supplementary material for: SETD7 Regulates the Differentiation of Human Embryonic Stem Cells
Source: PLoS One. 2016 Feb 18;11(2):e0149502. doi: 10.1371/journal.pone.0149502 (PMC4758617; doi:10.1371/journal.pone.0149502)

S5_Fig

A

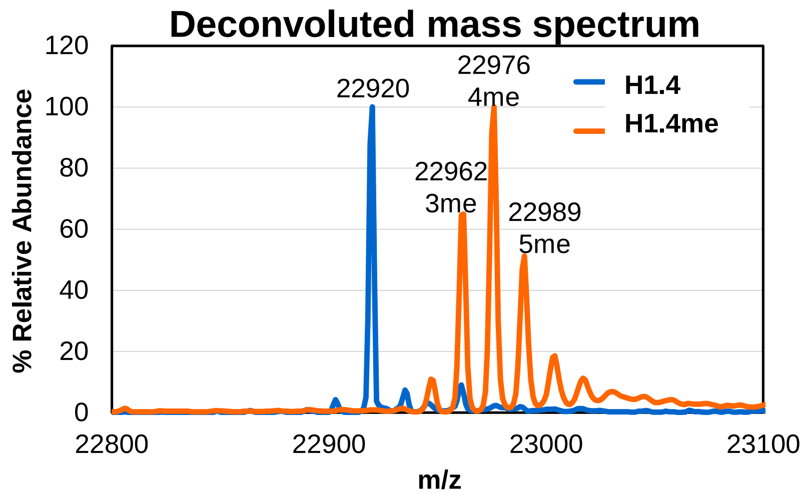

B

Assignment	H1.4				H1.4m			
	Buffer		DNA r=0.7		Buffer		DNA r=0.7	
	Band cm^{-1}	%	Band cm^{-1}	%	Band cm^{-1}	%	Band cm^{-1}	%
Turns			1685	3				
Turns	1670	17	1675	16	1673	13	1674	8
Turns	1660	17	1663	14	1662	15	1662	16
α-helix	1650	20	1649	27	1652	20	1652	22
Random coil/flexible regions	1640	24	1640	14	1642	27	1641	21
β-sheet	1630	10	1631	12	1630	21	1631	22
Low frequency β-sheet	1620	12	1620	14	1617	4	1618	11

C

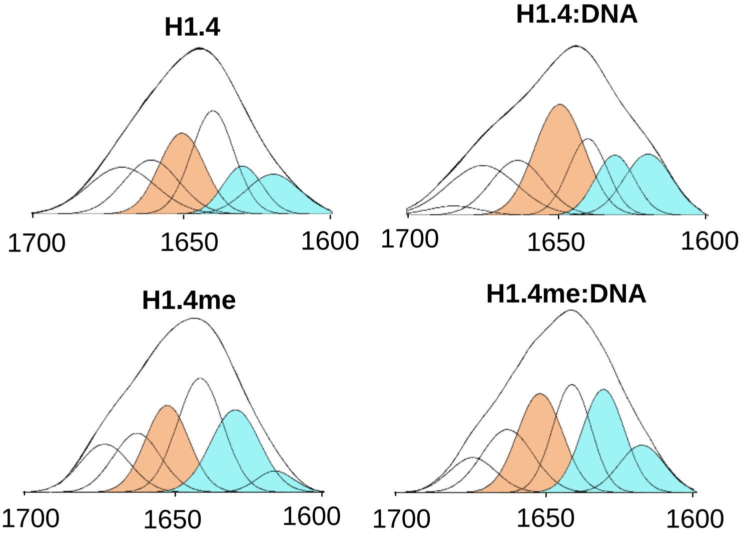

Supplement: S5 Fig — (A) Effect of methylation by SETD7 in the seconday structure of H1.4 in solution and bound to DNA. A, Mass spectrometry spectra of in vitro methylated H1.4 with 4 methyl groups incorporated on average, compared to unmethylated protein. (B) Infrared spectroscopy results for the unmethylated and methylated proteins in solution and bound to DNA. (C) Amide I decomposition of the unmethylated and methylated H1.4 in solution and bound to DNA. The α-helix component is highlighted in orange and the β-structure component is highlighted in light blue. Infrared measurements were performed at a protein concentration of 5 mg/ml in 10mM Hepes pH 7.0, plus 140 mM NaCl as described in Experimental Procedures. The protein/DNA ratio (r) (w/w) was 0.7. (PDF) [file pone.0149502.s005.pdf]
